# Decompressive craniotomy: an international survey of practice

**DOI:** 10.1007/s00701-021-04783-6

**Published:** 2021-03-18

**Authors:** Midhun Mohan, Hugo Layard Horsfall, Davi Jorge Fontoura Solla, Faith C. Robertson, Amos O. Adeleye, Tsegazeab Laeke Teklemariam, Muhammad Mukhtar Khan, Franco Servadei, Tariq Khan, Claire Karekezi, Andres M. Rubiano, Peter J. Hutchinson, Wellingson Silva Paiva, Angelos G. Kolias, B. Indira Devi, on behalf of the NIHR Global Health Research Group on Neurotrauma

**Affiliations:** 1 Division of Neurosurgery, Department of Clinical Neurosciences, Addenbrooke’s Hospital and University of Cambridge, Cambridge Biomedical Campus, Box 167, Cambridge, CB2 0QQ UK; 2 NIHR Global Health Research Group on Neurotrauma, University of Cambridge, Cambridge, UK; 3 Department of Neurosurgery, University of São Paulo, São Paulo, Brazil; 4 Department of Neurosurgery, Massachusetts General Hospital, Boston, MA USA; 5 Division of Neurological Surgery, Department of Surgery, College of Medicine, University of Ibadan, Ibadan, Nigeria; 6 Department of Neurological Surgery, University College Hospital, Ibadan, Nigeria; 7 Addis Ababa University, College of Health Science, Addis Ababa, Ethiopia; 8 Department of Neurosurgery, North West General Hospital and Research Center, Peshawar, Pakistan; 9 Department of Neurosurgery, Humanitas University, Department of Biomedical Sciences and Humanitas Clinical and Research Center- IRCCS, Milan, Italy; 10 Neurosurgery Unit, Department of Surgery, Rwanda Military Hospital, Kigali, Rwanda; 11 INUB/MEDITECH Research Group, El Bosque University, Bogota, Colombia; 12 MEDITECH Foundation, Clinical Research, Cali, Colombia; 13 Department of Neurosurgery, National Institute for Mental Health and Neurosciences, Bangalore, India

**Keywords:** Neurosurgery, Decompressive craniectomy, Decompressive craniotomy, Hinge craniotomy, Floating craniotomy, Traumatic brain injury, Stroke

## Abstract

**Background:**

Traumatic brain injury (TBI) and stroke have devastating consequences and are major global public health issues. For patients that require a cerebral decompression after suffering a TBI or stroke, a decompressive craniectomy (DC) is the most commonly performed operation. However, retrospective non-randomized studies suggest that a decompressive craniotomy (DCO; also known as hinge or floating craniotomy), where a bone flap is replaced but not rigidly fixed, has comparable outcomes to DC. The primary aim of this project was to understand the current extent of usage of DC and DCO for TBI and stroke worldwide.

**Method:**

A questionnaire was designed and disseminated globally via emailing lists and social media to practicing neurosurgeons between June and November 2019.

**Results:**

We received 208 responses from 60 countries [40 low- and middle-income countries (LMICs)]. DC is used more frequently than DCO, however, about one-quarter of respondents are using a DCO in more than 25% of their patients. The three top indications for a DCO were an acute subdural hematoma (ASDH) and a GCS of 9-12, ASDH with contusions and a GCS of 3-8, and ASDH with contusions and a GCS of 9-12. There were 8 DCO techniques used with the majority (60/125) loosely tying sutures to the bone flap. The majority (82%) stated that they were interested in collaborating on a randomized trial of DCO vs. DC.

**Conclusion:**

Our results show that DCO is a procedure carried out for TBI and stroke, especially in LMICs, and most commonly for an ASDH. The majority of the respondents were interested in collaborating on a is a future randomized trial.

**Supplementary Information:**

The online version contains supplementary material available at 10.1007/s00701-021-04783-6.

## Introduction

Traumatic brain injury (TBI) and stroke have devastating consequences and are major global public health problems. TBI is the largest contributor of death and disability among all trauma-related injuries [19], while stroke is the second leading cause of death worldwide [8], and the most common cause of acquired disability in adults [7]. Thus, even marginal improvements in the outcomes of these conditions can have profound implications, both at the level of the individual, as well as their family and the economy.

There is conflicting evidence regarding the efficacy of decompressive craniectomy in both TBI and stroke. The DECRA study [5] demonstrated that bifrontal decompressive craniectomy (DC) increases unfavorable outcomes in patients with mild intracranial hypertension, while the RESCUEicp trial [11] showed that, compared to continuing medical therapy, DC (bifrontal or unilateral) increases survival but also increases the rates of vegetative state, lower (dependent) and upper (independent at home) severe disability for patients with severe and refractory intracranial hypertension, compared to continuing medical therapy. In terms of stroke, pooled analysis from seven randomized trials demonstrate that decompressive surgery provides large reductions in mortality. However, many of those who survive tend to be disabled and require assistance with most basic needs [4]. Furthermore, the morbidity and complications arising from a cranioplasty post-DC cannot be ignored.

The burden of TBI and stroke in low- and middle-income countries (LMICs) is profound. The vast majority of neurotrauma [6] as well as 63% of all ischemic and 80% of all hemorrhagic strokes occur in LMICs [15]. However, it is not clear if the evidence from these DC trials can be naturally extrapolated to centers based in LMICs. Less than 10% percent of patients recruited for the RESCUEicp trial [11] were from LMICs, while the DECRA trial [5] did not include any patients from LMICs. For stroke, the three major trials DECIMAL [20], DESTINY [12], and HAMLET [10] were all conducted solely in high-income countries.

Up to a quarter of individuals who undergo a surgical procedure will be subject to financial catastrophe as a direct result of seeking help, and this financial burden is most pronounced in LMICs [17]. Specialities such as neurosurgery are often considered a luxury in some LMICs, and neurosurgical services can be expensive and difficult to access [2, 3]. To address this, interventions that may not be considered as a priority in high-income countries need to be revisited as a potential cost-effective measure. A potential alternative to traditional DC that may be applicable especially in LMICs is a floating or hinge craniotomy (HC) [14]. The procedure is performed by either letting the bone flap “float” [9], prior to closing the skin or by securing it in a non-circumferential manner thus creating a hinge, which would still allow for some cerebral expansion through the hinge defect [1]. Importantly, the use of HC would drastically reduce or entirely remove the prospect of a subsequent cranioplasty, thus, reducing the financial implications of a second operation and the potential complications that it entails. However, the evidence on this technique is limited and is largely retrospective and of low quality [14]. We recently established the National Institute of Health Research (NIHR) Global Health Research Group on Neurotrauma with the primary aim to improve the care of patients with TBI in LMICs. As a project from this group, we first published a scoping review on this topic to understand the current evidence base of hinge craniotomy [16]. We introduced the term decompressive craniotomy (DCO), which will be used in the rest of this paper. A DCO incorporates any technique where the bone flap is replaced but not rigidly fixed (e.g., floating or hinged).

We believed that a survey examining the current practice of DCO worldwide with particular emphasis on participation from LMICs would allow us to understand how DCO is currently being used. This survey was undertaken in collaboration with the Neurotrauma Committee of the World Federation of Neurosurgical Societies (WFNS). The primary aim of the survey was to understand the current extent of usage of DC and DCO for TBI and stroke worldwide. The secondary aims were (i) to explore the indications and technical variations with regards to DCO and (ii) understand if there is interest from centers worldwide to participate in a randomized trial comparing DC versus DCO.

## Materials and methods

### Development and approval

A questionnaire was developed by members of the NIHR Global Health Research Group on Neurotrauma and the Neurotrauma Committee of the WFNS (Electronic Supplementary Material). The questionnaire was piloted and modified based on feedback by members of the writing group of this paper. The Neurotrauma Committee of the WFNS approved the survey content and subsequent dissemination.

### Dissemination

A secure online survey tool (Google Forms**®**) was used to disseminate the questionnaire to clinicians worldwide involved in the operative management of patients with TBI. Respondents comprised a non-probabilistic sample of neurosurgeons invited through the electronic mailing lists of various neurosurgical societies, email to personal contacts, and social media platforms (Twitter, Facebook, and WhatsApp). The WFNS provided a link to the survey on their email newsletter. The survey was open between 21st June 2019 and 21st November 2019.

### Eligibility

Those eligible to complete the survey were surgeons involved in the operative management of patients with TBI. Clinicians involved solely in the medical management of TBI (intensive care and emergency physicians) were excluded from participating due to the inherent technical and operative questions that were asked.

### Analysis

All data acquired was kept confidential on a secure online platform. No patient information was requested. It was not possible to ascertain response rates due to the wide dissemination of the survey via several channels. Duplicates were removed via matching those of an identical name and hospital. Descriptive analysis was undertaken.

## Results

### Demographics

**Fig. 1:**
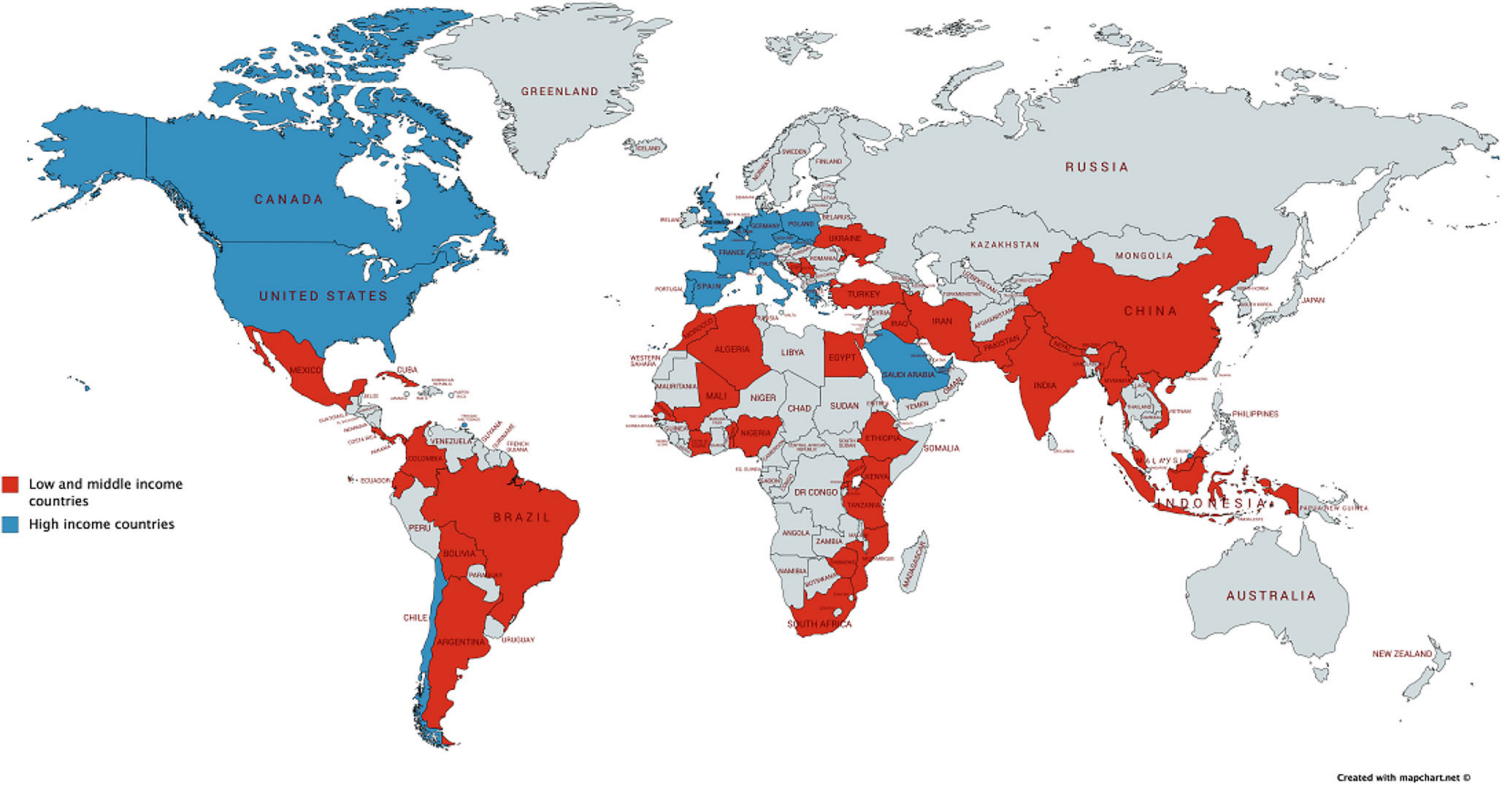
Responses from countries surveyed split by low and middle income and high income (208 responses)

The survey was open for participation for 153 days. We received 208 responses; 58% (*n*=120) were from consultants/attendings, 20% (*n*=41) from senior trainees/chief resident/senior fellows, and 23% (*n*=47) from more junior trainees/residents. All participants were neurosurgeons or neurosurgical trainees, and we received responses from 60 countries (40 LMICs). Just over three quarters of responses (*n*=160, 77%) were from surgeons residing in LMICs, and 48% of the total responses from LMICs were from 4 countries (India, Brazil, Colombia, and Pakistan). Figure 1 shows the responses worldwide.

### Setting

Of those surveyed, 21% (*n*= 44) practiced in a private setting, while 79% (*n*=164) practiced in a public hospital.

### Indications

**Fig. 2:**
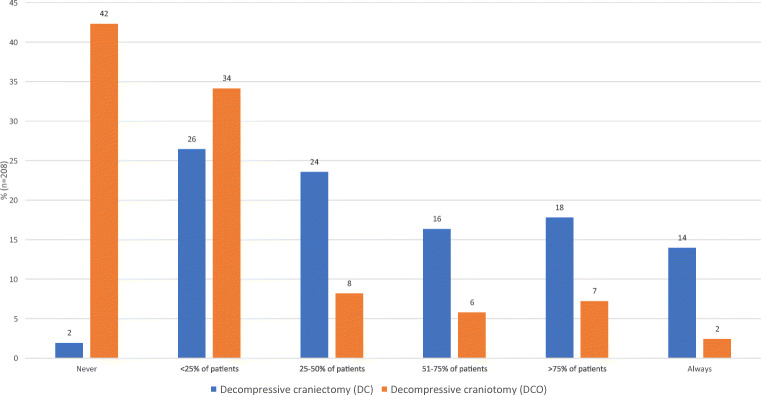
In your practice, do you ever use the technique of decompressive craniectomy (DC) (i.e., bone flap left out or stored in a subcutaneous pocket) for patients with TBI or stroke (large volume ischaemic stroke or haemorrhagic)?” and in your practice, do you ever use the technique of decompressive craniotomy (DCO) (i.e., bone flap replaced but not rigidly fixed) for patients with TBI or stroke (large volume ischaemic stroke or haemorrhagic)? (*n*=208)

**Fig. 3:**
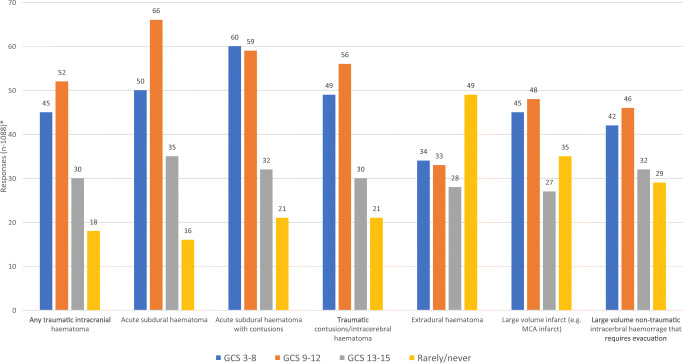
In which of the following situations, do you tend to use decompressive craniotomy (DCO) in your practice? *Responders could select multiple categories

Overall, DC appears to be used more frequently compared to DCO. However, about one-quarter of respondents are using a DCO in more than 25% of their patients. Only 2% (4/208) stated they never perform DC compared to 42% (87/208) for DCO (Fig. 2). When only respondents from LMICs were included, 32% (51/160) stated they never perform a DCO. Taking the pre-operative GCS state and the specific TBI into account, the three most common indications for a DCO were an acute subdural hematoma and a GCS of 9-12, acute subdural hematoma with contusions and a GCS of 3-8, and acute subdural hematoma with contusions and a GCS of 9-12. Extradural hematoma appeared to be the least frequent indication. DCO was also being performed in patients whose neurology was of a good grade (GCS 13-15) (Fig. 3).

### Procedure

**Fig. 4:**
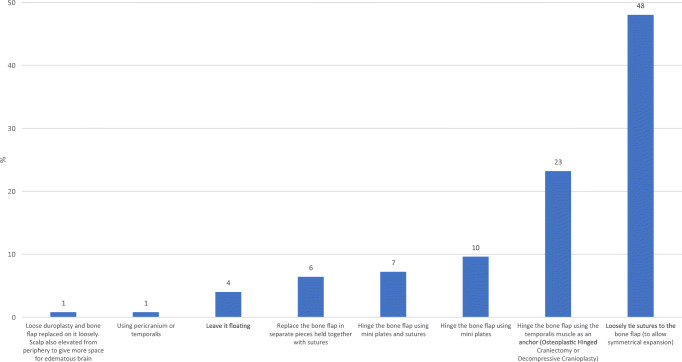
Which is your preferred decompressive craniotomy (DCO) technique? (*n*=125)

**Fig. 5:**
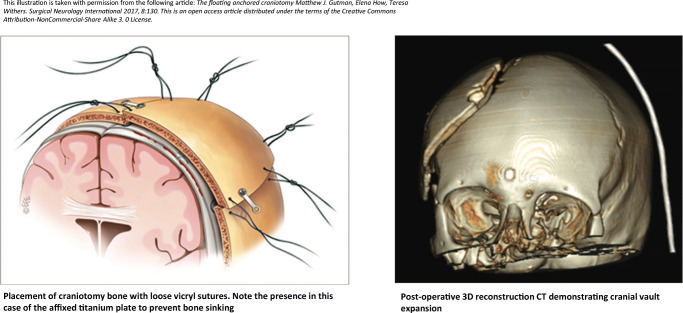
Illustration to demonstrate the technique of loosely tying sutures in decompressive craniotomy (DCO)

**Fig. 6:**
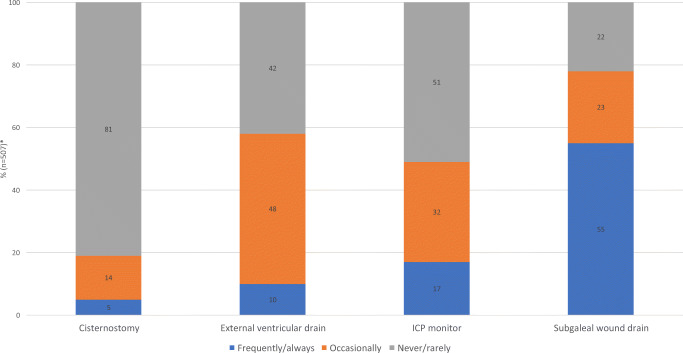
Do you tend to supplement a decompressive craniotomy (DCO) for TBI/stroke with any of the following? *Responders could select multiple categories but within each category, the options are self excluding, thus, aiding calculation of a percentage per category

There were 8 DCO techniques in use with the majority [48% (60/125)] loosely tying sutures to the bone flap (Figs. 4 and 5). Eighty-one percent (101/125) never thinned the inner table of the bone flap, while 18% (22/125) did so occasionally. There were nine techniques on how to manage the dura, with 52% (63/121) undertaking a durotomy followed by sutured duraplasty (with dural substitute or autologous graft), 24% (29/121) undertaking a durotomy and leaving the dura open (exposed brain tissue and dura covered with absorbable hemostatic material), and 19% (23/121) performing a durotomy followed by a simple onlay duraplasty (with dural substitute or autologous graft). The most common adjunct or supplement to the DCO was a subgaleal wound drain and the least common was a cisternostomy. The results also show that up to 49% insert an ICP monitor when undertaking a DCO (17% frequently/always, 32% occasionally) (Fig. 6).

### Post-operative destination

For the question “what is the usual destination of patients undergoing decompressive craniotomy post-operatively, 58% (73/125) stated ICU, 26% (32/125) stated ICU but beds are not always available, 13% (16/125) ICU or ward depending on patient’s pre-operative GCS, and 3% (4/125) neurosurgical ward since there is no ICU.

### Interest in collaborating in a randomized trial of DC vs. DCO

From 208 responses, the majority (82%) stated that they were interested in participating, while only 6% stated that they were not. The proposed annual recruitment rate from 196 responses ranged from 0 to 500 with a mean of 41 patients.

## Discussion

### Uncertainty

Our results show that DC is a common technique for patients with TBI and stroke used by 98% of respondents worldwide. However, more than half (58%) have also used the DCO procedure. When including only responses from LMICs, the figure is higher with 68% of surgeons having used DCO. These results are not surprising. DC is a procedure taught early in neurosurgical training, is well established and researched, with multiple randomized trials in existence examining its efficacy [5, 10–12]. It is also a procedure that is proven to be efficacious in reducing intracranial pressure (ICP), which is an immediate concern when faced with a neurologically deteriorating patient post-TBI or stroke. The assumption that DCO provides inadequate control of ICP is likely the most common reason for its lower popularity. However, several studies have shown that DCO is comparable to DC in the control of ICP. Kano et al. undertook a seven-year study comparing DCO and DC in 58 patients with TBI and stroke [13]. They found no difference in the post-operative mRS or GOS between the two groups. Furthermore, six patients in their DC arm developed a bone flap infection compared to zero patients in the DCO group. However, 2 patients in their DCO group had to have a subsequent DC due to elevated ICP. The authors concluded the use of DCO with subsequent ICP monitoring could be a possible alternative to DC. Their suggestion seems to reflect real-world practice and concurs with the results of our survey. The study by Kenning et al. also concluded that DCO and DC are comparable in terms of control of ICP and post-operative functional outcomes [14]. A recent extensive scoping review on the topic of DCO provides preliminary evidence that there is clinical equipoise on the effectiveness of DC versus DCO [16]. Nevertheless, the current evidence is of low quality and non-randomized, thus, higher impact studies are warranted.

### Indications

Our results suggest that the presence of an acute subdural hematoma seems to be the most common reason for undertaking a DCO, while an extradural hematoma being the least common. This finding mirrors the indications of DC, since a DC is often carried out for acute subdural hematomas and rarely performed for extradural hematomas, since in isolated extradural hematomas, the underlying brain parenchyma is infrequently injured. A GCS between 9 and 12 seems to be the most common GCS category where a DCO is undertaken, in five out of the seven pathologies surveyed. The use of DCO seemed to be lower among most pathologies when the patient’s GCS was poor (GCS 3-8) compared to a GCS of 9-12. This could be due to the uncertainty faced by the surgeon of the DCO’s effectiveness in successfully reducing ICP. As aforementioned, this uncertainty is likely to be multifactorial, however, the limited evidence available states this technique may actually be comparable to DC. Interestingly, DCO was also being performed in patients with a good neurological grade (GCS 13-15). The reason for this is unclear. It may be due to resource poor centers not having the infrastructure to monitor the patient closely post TBI or stroke for neurological deterioration and thus the DCO is undertaken in an almost prophylactic manner. This specific area warrants further investigation.

### Techniques

Eight different techniques were utilized by respondents. The majority opted to loosely tie the bone flap in place with sutures, and other techniques were utilized such as hinging the bone flap to the temporalis muscle. There are currently no studies investigating the superiority of one technique when re-attaching the bone in a DCO. Even if DCO becomes mainstream, we believe the techniques used to re-attach the bone are likely to vary widely depending on surgeon preference and resources available, but nevertheless, it may be an area of further research.

### Interest in a randomized trial

The survey shows that DCO is practiced more in resource poor nations. This again could simply be due to cost. A second operation, even with the use of autologous bone, could bankrupt a family residing in a country without universal health coverage resulting in the patient never receiving a cranioplasty. A permanent cranial defect will further impact the patient’s ability to rehabilitate and get back to being a functioning member of society, thus, compounding the financial implications [18]. Since TBI and stroke are more common in LMICs, this has serious economic consequences [6, 15]. Our results show that DCO is a procedure practiced in LMICs and that there is interest in collaborating in a randomized trial. If DCO is proven to be superior or at least on par with outcomes provided by DC, this is likely to be a landmark finding with significant economic advantages. The prospect of reducing the number of operations required post-TBI or stroke from two to one could be generalizable to any neurosurgical setting globally, due to the inherent reductions in cost, surgical resources, infection rates, and operative risk.

### Limitations

This survey has a number of limitations. First, the survey was disseminated via several channels, thus, it was not possible to ascertain response rates. However, we believe our 208 responses from 60 countries provide a pragmatic overview on the current global practice. Second, there is a risk of bias around the question pertaining to the interest in a trial on this topic. This is because individuals who completed the questionnaire are from a limited potentially self-selected group of neurosurgeons interested in a niche question. Third, we did not compare if DCO was preferred to DC for certain indications. This was an intentional omission to maintain brevity since the primary objective was not to compare the two procedures. Fourth, we did not evaluate if other factors such as a patient’s ability to pay or cost of the procedure factored into the decision making of the operation chosen. Last, since the survey was web-based, a pre-requisite was that the potential audience has access to the internet. This might not have been the case in the most rural and resource poor centers.

## Conclusion

There is a pressing need for cost-effective solutions for the surgical treatment of patients with TBI and stroke, especially in LMICs where the disease burden is greatest. Decompressive craniotomy is a procedure carried out for TBI and stroke, especially in LMICs, and most commonly for an acute subdural hematoma. Several techniques are currently being used to re-attach the bone flap back on the cranium, with loosely tying sutures to the bone flap being the most common. There is an interest globally among the respondents spanning 60 countries (42 LMICs) in participating in a randomized trial comparing DC versus DCO.

## Supplementary Information

ESM 1 (DOCX 21 kb)

## Data Availability

Raw data available on request.
