# Supplementary material for: Decompressive craniotomy: an international survey of practice
Source: Acta Neurochir (Wien). 2021 Mar 18;163(5):1415–22. doi: 10.1007/s00701-021-04783-6 (PMC8053664; doi:10.1007/s00701-021-04783-6)
Supplement: Supplementary file 1 — (DOCX 21 kb) [file 701_2021_4783_MOESM1_ESM.docx]

**Decompressive craniotomy: an international survey of practice**

1. **Grade**

Consultant / Attending

Senior Registrar / Chief Resident / Senior Fellow

Trainee / Resident

1. **Speciality** (Please kindly DO NOT fill this survey if you are NOT involved in the operative management of patients with TBI / stroke)

Neurosurgeon

A non-neurosurgeon who performs cranial neurosurgery

Other

1. **Country of hospital**
2. **Setting of your practice**

Public hospital covering a predominantly urban area

Private hospital covering a predominantly urban area

Public hospital covering a predominantly rural area

Private hospital covering a predominantly rural area

Other

1. **In your practice, do you ever use the technique of DECOMPRESSIVE CRANIECTOMY (i.e. bone flap left out or stored in a subcutaneous pocket) for patients with TBI or stroke (large volume ischaemic stroke or haemorrhagic)?**

Never

To note, we introduced the term decompressive craniotomy (DCO) in the paper. A DCO incorporates any technique where the bone flap is replaced but not rigidly fixed (e.g. floating, hinged).

<25% of patients

25-50% of patients

51-75% of patients

>75% of patients

Always

Other

1. **In your practice, do you ever use the technique of HINGE / FLOATING CRANIOTOMY (i.e. bone flap replaced but not rigidly fixed) for patients with TBI or stroke (large volume ischaemic stroke or haemorrhagic)?**

Never

<25% of patients

25-50% of patients

51-75% of patients

>75% of patients

Always

Other

1. **In which of the following situations, do you tend to use a hinge / floating craniotomy in your practice (you can select as many as you like)?**

|  | GCS 3-8 | GCS 9-12 | GCS 13-15 | Rarely / never |
| --- | --- | --- | --- | --- |
| Any type of traumatic intracranial haematoma |  |  |  |  |
| Acute subdural haematoma |  |  |  |  |
| Acute subdural haematoma with contusions |  |  |  |  |
| Traumatic contusions / intracerebral haematoma |  |  |  |  |
| Extradural haematoma |  |  |  |  |
| Large volume infarction (e.g. MCA infarct) |  |  |  |  |
| Large volume non-traumatic intracerebral haemorrhage that requires evacuation |  |  |  |  |

1. **Which is your preferred hinge / floating craniotomy technique?**

Hinge the bone flap using mini plates

Hinge the bone flap using mini plates and sutures

Hinge the bone flap using the temporalis muscle as an anchor (Osteoplastic Hinged Craniectomy or Decompressive Cranioplasty)

Loosely tie sutures to the bone flap (to allow symmetrical expansion)

Replace the bone flap in separate pieces held together with sutures

1. **Do you tend to thin the inner table of the bone flap to gain additional volume?**

Never / rarely

Occasionally

Frequently / always

Always

1. **How do you manage the dura?**

Durotomy undertaken and dura left open (exposed brain tissue and dura covered with absorbable haemostatic material, such as Surgicel)

Durotomy followed by sutured duraplasty (with dural substitute or autologous)

Durotomy followed by simple onlay duraplasty (with dural substitute or autologous)

Dura scarified

Other

1. **Do you tend to supplement a hinge / floating craniotomy for TBI / stroke with any of the following?**

|  | Never / rarely | Occasionally | Frequently / always |
| --- | --- | --- | --- |
| Cisternostomy |  |  |  |
| External ventricular drain |  |  |  |
| ICP monitor |  |  |  |
| Subgaleal wound drain |  |  |  |

1. **In your practice, what is the usual destination of patients undergoing hinge / floating craniotomy post-operatively?**

ICU – always

ICU - but beds are not always available

Neurosurgical ward - as we do not have ICU

ICU or ward - depending on patient's pre-operative GCS

Other

1. Would you be interested in collaborating on a proposed randomised trial comparing decompressive craniectomy versus hinge / floating craniotomy for TBI / stroke?

Unsure / neutral

Probably yes

Absolutely

1. **Of the patients you manage in 1 year, how many do you think would be eligible for randomisation in such a trial? Please provide a NUMBER (not percentage).**
2. **Name**
3. **Hospital**
4. **Your email address**
